# Head-to-head comparison of a Si-photomultiplier-based and a conventional photomultiplier-based PET-CT system

**DOI:** 10.1186/s40658-021-00366-7

**Published:** 2021-02-25

**Authors:** Jenny Oddstig, Gustav Brolin, Elin Trägårdh, David Minarik

**Affiliations:** 1 Radiation Physics, Department of Hematology, Oncology and Radiation Physics, Skåne University Hospital, 221 85 Lund, Sweden; 2 Clinical Physiology and Nuclear Medicine, Skåne University Hospital and Lund University, Carl Bertil Laurells gata 9, Skåne University Hospital, 205 02 Malmö, Sweden; 3 Wallenberg Center for Molecular Medicine, Lund University, Carl Bertil Laurells gata 9, Skåne University Hospital, 205 02 Malmö, Sweden

**Keywords:** PET/CT, SiPM, Analogue PM-tubes, Phantom measurements

## Abstract

**Background:**

A novel generation of PET scanners based on silicon (Si)-photomultiplier (PM) technology has recently been introduced. Concurrently, there has been development of new reconstruction methods aimed at increasing the detectability of small lesions without increasing image noise. The combination of new detector technologies and new reconstruction algorithms has been found to increase image quality. However, it is unknown to what extent the demonstrated improvement of image quality is due to scanner hardware development or improved reconstruction algorithms. To isolate the contribution of the hardware, this study aimed to compare the ability to detect small hotspots in phantoms using the latest generation SiPM-based PET/CT scanner (GE Discovery MI) relative to conventional PM-based PET/CT scanner (GE Discovery 690), using identical reconstruction protocols.

**Materials and methods:**

Two different phantoms (NEMA body and Jasczcak) with fillable spheres (31 μl to 26.5 ml) and varying sphere-to-background-ratios (SBR) were scanned in one bed position for 15–600 s on both scanners. The data were reconstructed using identical reconstruction parameters on both scanners.

The recovery-coefficient (RC), noise level, contrast (sphere_peak_/background_peak_-value), and detectability of each sphere were calculated and compared between the scanners at each acquisition time.

**Results:**

The RC-curves for the NEMA phantom were near-identical for both scanners at SBR 10:1. For smaller spheres in the Jaszczak phantom, the contrast was 1.22 higher for the DMI scanner at SBR 15:1. The ratio decreased for lower SBR, with a ratio of 1.03 at SBR 3.85:1. Regarding the detectability of spheres, the sensitivity was 98% and 88% for the DMI and D690, respectively, for SBR 15:1. For SBR 7.5, the sensitivity was 75% and 83% for the DMI and D690, respectively. For SBR 3.85:1, the sensitivity was 43% and 30% for the DMI and D690, respectively.

**Conclusion:**

Marginally higher contrast in small spheres was seen for the SiPM-based scanner but there was no significant difference in detectability between the scanners. It was difficult to detect differences between the scanners, suggesting that the SiPM-based detectors are not the primary reason for improved image quality.

## Background

Recently, a novel generation of PET scanners, with silicon (Si)-photomultiplier (PM) technology replacing the conventional photo-multiplier tubes (PMTs) [1–3], was introduced and marketed as “digital PET” systems, with promises of improved spatial resolution, lesion detectability, counting performance, and potential reduction of activity and scan time. Concurrent with the introduction of this new generation of scanners was the development of new reconstructions methods to increase the detection of small lesions without increasing image noise. An example is the block-sequential regularisation expectation algorithm (BSREM) (incorporated in Q.Clear GE Healthcare, Milwaukee, WI, USA) [4]. The combination of new detector technology and new reconstruction algorithms have been found to increase image quality, increase the maximum standardised uptake values (SUV) in lesions, and increase the lesion-to-blood-pool SUV ratios in smaller lesions and number of detected lesions compared to the previous generation of scanners [5–9]. However, it is currently unknown to what extent the improved image quality should be attributed to the new detector design versus improved reconstruction algorithms. Previous studies comparing scanners with SiPM-technology to conventional analogue PET/CT-systems have studied phantoms with large spheres or brain phantom [3, 10]. To our knowledge, no study has used phantoms with small spheres below 500 μl. This study aimed to compare the ability to detect small hotspots in phantoms using a SiPM-based and a conventional PM-based PET/CT scanner from the same vendor, located in the same department. The head-to-head comparison was performed using the same phantom with identical acquisition and reconstruction protocols, thus eliminating the uncertainty introduced by different phantom preparations and the impact of different reconstruction algorithms.

## Material and methods

### PET/CT systems

A Discovery D690 (D690) PET/CT installed in 2011 and a Discovery MI (DMI) PET/CT installed in 2017 (both GE Healthcare, Milwaukee, WI, USA) were used for image acquisition in this study. The D690 is a three-ring scanner with LYSO crystals and PMTs combined with a 64-slice CT scanner (details are summarised in Table 1). The DMI has a configuration of four rings of LYSO crystals and silicon photomultipliers (SiPM) combined with a 128-slice CT [1]. Both PET/CT scanners are cross-calibrated to the same dose calibrator, and the calibration is validated monthly using a uniform cylindrical phantom filled with ^18^F-FDG.

**Table 1:** Characteristics of the Discovery MI and Discovery 690 scanners

PET/CT system	Discovery MI	Discovery PET/CT 690
Crystal material	LYSO/LBS	LYSO/LBS
Light detection	SiPM	PMT
Number of detector rings	4	3
Number of crystals	19584	13824
Size of crystals [mm^3^]	3.95 × 5.3 × 25	4.2 × 6.3 × 25
Slice thickness [mm]	2.79	3.27
Axial FOV [mm]	200	157
FOV [mm]	700	700
Overlap [%]^a^	23.9	23.4
NEMA Sensitivity [cps/kBq]	13.8	6.9
Image planes in the axial FOV	71	47
FWHM axial @1 cm [mm]	4.7	5.2
Coincidence window width (ns)	4.9	4.9
Coincidence timing resolution (ps)	375.4	544.3
Lower energy threshold (keV)	425	425

^a^The default overlap in the scanners. The overlap can be adjusted by the user

### Phantom studies

The two PET/CT systems are in close vicinity of each other, which makes it possible to compare the performance of the scanner with the same phantom from the same phantom preparation. The phantom measurements were carried out with ^18^F-FDG and two different phantoms with fillable spheres:

- The NEMA IEC Body Phantom (Data Spectrum Corporation, Durham, NC, USA) with fillable spheres was used. The spheres have a specified inner diameter of 10, 13, 17, 22, 28, and 37 mm and were filled with ^18^F-FDG. The volume of the phantom is 9.7 L. The sphere-to-background ratio (SBR) was 10:1, and the activity concentration was approximately 20 kBq/ml in the spheres at the time of measurement.
- The cylindrical Jaszczak phantom (Data Spectrum Corporation, Durham, NC, USA) (diameter of 21.6 cm and a volume of 6.9 L) with microspheres (4.0, 5.0, 6.2, 9.9, 11.9, and 14.4 mm diameter) was used. The phantom was prepared and scanned on three different occasions, in which the SBR for the four smallest spheres was 15:1, 7.5:1, and 3.75:1, to simulate small hot spots in different tissues representing, e.g. tumours in the lung (15:1) and liver (3.75:1). The activity concentration in the spheres was approximately 85 kBq/ml at the time of measurement for all three SBR preparations. The two larger spheres were filled with water instead of activity.

PET acquisitions were performed for all phantoms using a single bed position for 10 min on both camera systems, accompanied by CT acquisition for attenuation correction (120 kVp). The phantoms were positioned with the spheres in the centre of the axial field of view. Sinograms with 600, 300, 180, 150, 120, 90, 60, 45, 30, and 15 s/bed were created from the acquired list-mode data. For the second acquisition (after moving the phantom from the first to the second scanner), the acquisition time was prolonged to account for physical decay of ^18^F and thereby ensuring an equal time-activity product for the two PET acquisitions. Image reconstruction was performed on the scanner console with identical reconstruction parameters for both systems, using OSEM with four iterations and 16 subsets, including point spread function modelling for resolution recovery, time-of-flight, and attenuation correction. The reconstructed transaxial matrix size was 256 × 256 with a pixel size of 2.7 × 2.7 mm^2^. No post-filtering was applied.

The measurements were repeated with a new phantom preparation in revised order.

One additional experiment was performed using a homogenous, cylindrical phantom with a 20 cm diameter, a length of 30 cm, and a volume of 9.4 L. The phantom was filled with ^18^F-FDG and water, with a resulting activity concentration of 6.5 kBq/ml. The phantom was scanned with two bed positions for 10 min per bed position on both PET/CT-cameras with an overlap of bed positions of 24% as recommended by the vendor. A CT for attenuation correction was also performed. Image reconstruction and correction for radioactive decay between the two scans was performed as described above.

### Image analysis

The recovery coefficient (RC) for the NEMA Body phantom was calculated according to the NEMA protocol [11], for reconstructions corresponding to 90 s and 600 s acquisition time, respectively. Five independent acquisitions/reconstruction were generated from the list-mode file for the 90s measurement. The RC for each sphere diameter was calculated as the mean value for the two phantoms for the 600 s measurement (*n* = 2) and for 5 reconstructions and the two phantoms for the 90 s measurement (*n* = 10). The coefficient of variation (COV) (i.e. the standard deviation divided by the mean value) was calculated in a ROI covering the whole background volume except a margin around the spheres and edge of the phantom. The mean COV from the two different phantom preparations was used (*n* = 2).

The sphere-to-background contrast was evaluated in both the NEMA and Jaszczak phantoms. The contrast was in this case defined as the ratio between the peak value in the sphere and the peak value in the background. This metric, we believe, can be better at reflecting the distinguishability between very small hot spheres and noise, as compared to standard contrast to noise ratio. The peak value was calculated as the mean value of a 3 × 3 × 3 voxel cube centred on the voxel with the highest activity concentration in the defined ROI. The ROIs were drawn in the axial image slice at the centre of the spheres in the PET image with 600 s acquisition time, and then copied to the PET series representing different acquisition times, which could lead to a different position of the cube depending on which voxel had the highest value inside the ROI. The peak value in the background was calculated around the background voxel with the highest activity concentration in a ROI covering the whole background except a margin around the spheres and edge of the phantom, in the same slice as the sphere ROIs.

The detectability of each sphere at each acquisition time and SBR was assessed by means of subjective grading on a binary scale. Spheres that were considered clearly visibly were assigned a score of 1, whereas spheres that were not visible or not distinguishable from background noise were given a score of zero. The sensitivity was calculated as the sum of the scores divided with the highest possible score. The evaluation was performed in consensus by two nuclear medicine physicists.

In the homogenous phantom, circular ROIs with a diameter of 5 cm were drawn in the centre of each slice and the COV was calculated as a function of slice position. The mean COV over ROIs in five slices was used.

### Statistical analysis

The data are presented as mean ± 1 standard deviation (SD).

Linear and logistic mixed models were employed to evaluate the difference between DMI and D690. In all models, sphere and time were included as random effects and SBR was added as a factor to each model. A *p* value below 0.05 indicates the statistical significance and all analyses were performed in Stata 16SE.

## Results

**Fig. 1:**
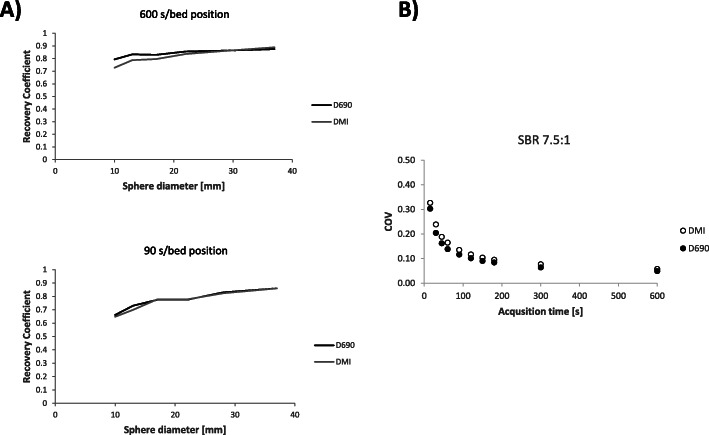
**a** The recovery curve (RC) for the NEMA Body phantom (sphere diameter 10–37 mm) with a sphere to background ratio (SBR) of 10:1 at 600 s and 90 s per bed position. The data presented is a mean value of two phantom preparations for the 600 s measurement, and for two phantom preparations and 5 independent acquisitions/reconstructions for the 90 s measurement. **b** The noise level [the coefficient of variation (COV)] in the background of the phantom at different times per bed position

The measured COV was higher for the DMI than for the D690 for all acquisition times studied (Fig. 1). The recovery coefficient measured in the NEMA Body phantom showed slight differences between the PET/CT systems at any acquisition time (noise level) and SBR studied (Fig. 1).

**Fig. 2:**
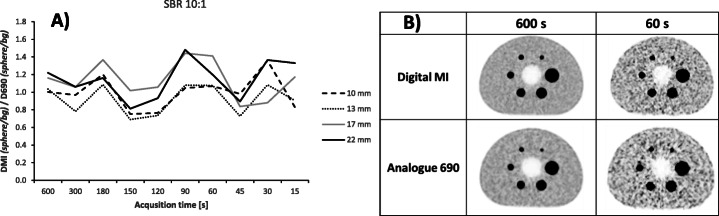
**a** The ratio between the Discovery MI (DMI) and the Discovery 690 (D690) of the sphere_peak_/background_peak_ ratio for acquisition times 600–15 s/bed in the NEMA body phantom. The hot spheres had diameters of 10–22 mm and the phantom was filled with SBR 10:1 with ^18^F-FDG. **b** Images of the phantom with 600 and 60 s per bed position at an SBR of 10:1

The ratio of the contrast (sphere_peak_/background_peak_)^DMI^/(sphere_peak_/background_peak_)^D690^ for the NEMA body phantom (sphere diameter 10–37 mm) was nearly one (1.05 ± 0.22) at SBR 10:1 and acquisition times 15–600 s (Fig. 2). The noise level in the background for an acquisition time of 600 s per bed was comparable with the noise level seen in the liver in patient FDG-scans.

**Fig. 3:**
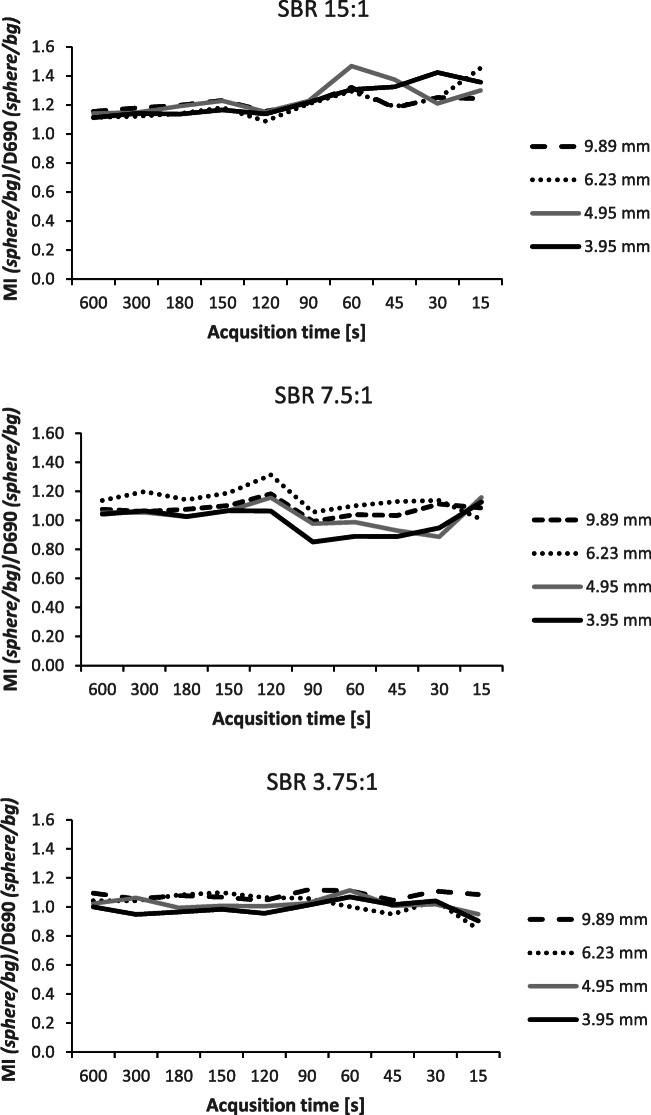
The ratio between the Discovery MI and the Discovery 690 of the sphere_peak_/background_peak_ ratio for acquisition times 600–15 s/bed in the cylindrical Jaszczak phantom. The spheres had diameters between 3.95 and 9.89 mm and the phantom was filled with ^18^F-FDG. The sphere-to-background ratios (SBRs) were 15:1, 7.5:1, and 3.75:1, respectively

**Fig. 4:**
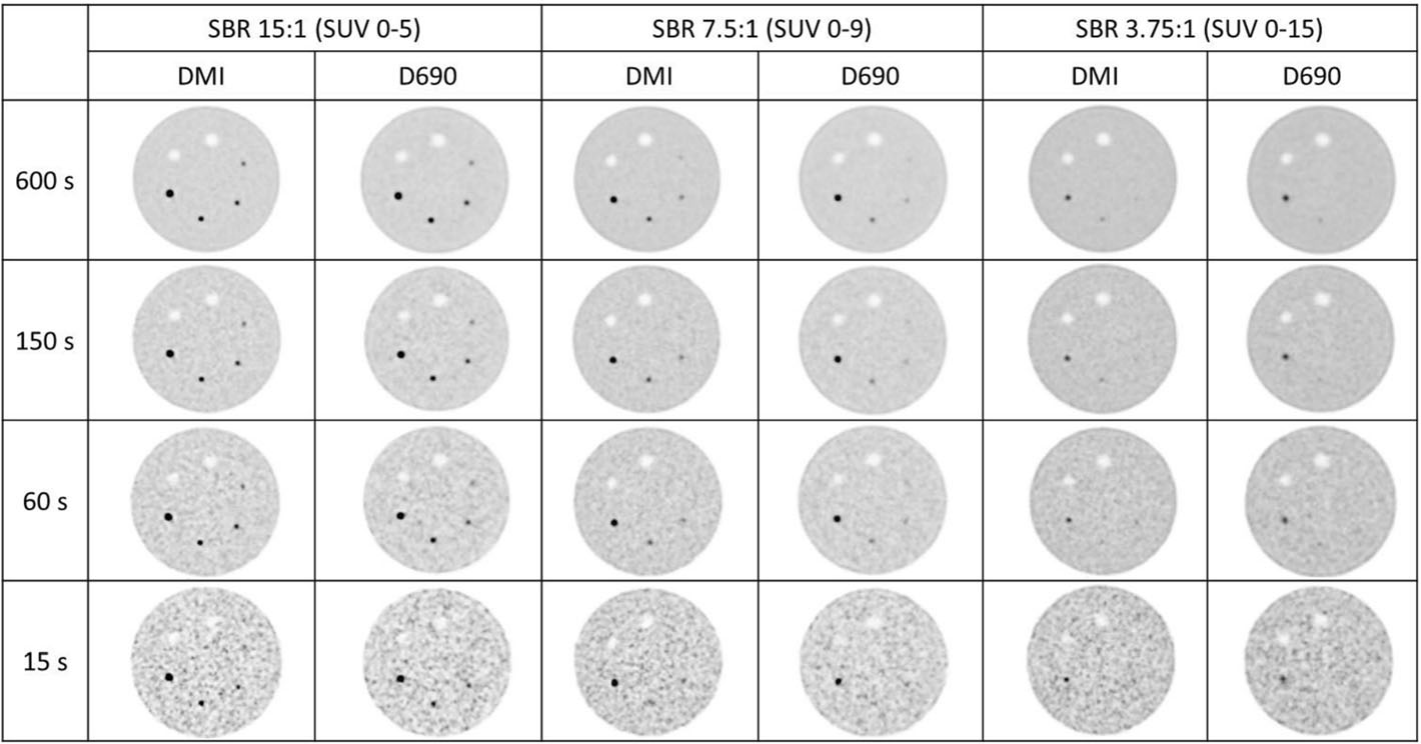
Example images of the Discovery DMI and Discovery 690 for different sphere-to-background ratios (SBRs) and acquisition times. The greyscale has been adjusted for each SBR experiment to facilitate visual comparison

The contrast for the small spheres (diameter 3.95–9.89 mm) in the Jaszczak phantom was, on average, 1.22 ± 0.10 times higher for the DMI compared to the D690 scanner at SBR 15:1 (Fig. 3). The ratio between the scanners decreased for lower SBR to 1.06 ± 0.10 and 1.03 ± 0.06 at SBR 7.5:1 and 3.85:1, respectively. The linear mixed models show that the contrast for the small spheres for all measure times and SBR was lower for the D690: − 0.283 (95% CI − 0.535, − 0.031). This difference is statistically significant. Example images of the DMI and D690 for different SBRs and acquisition time can be seen in Fig. 4.

**Fig. 5:**
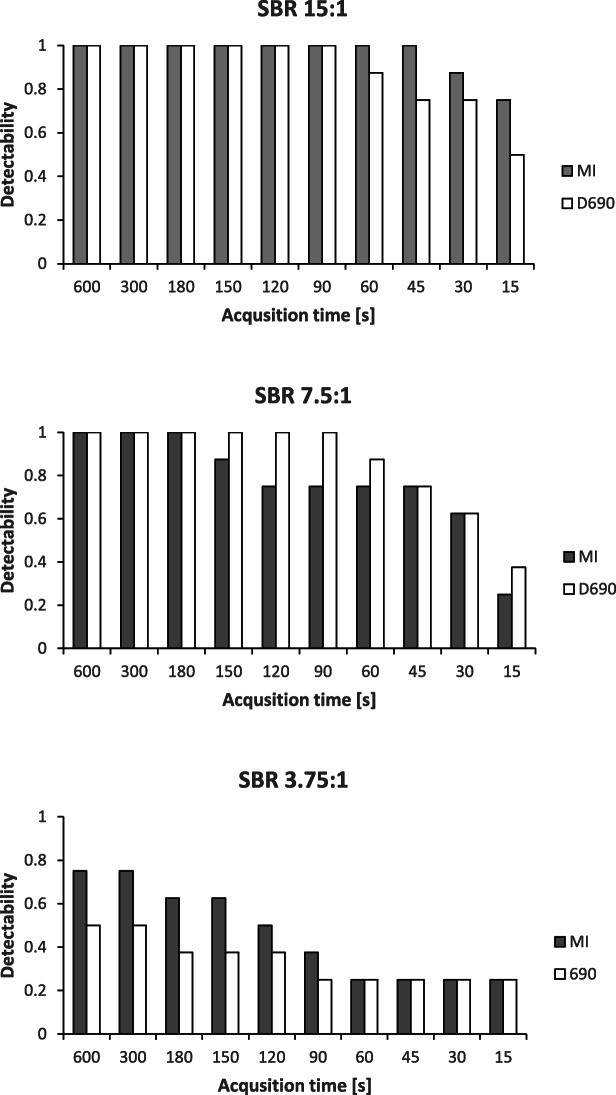
Detectability of the small spheres in the Jaszczak phantom at different sphere-to-background ratios (SBRs) and different acquisition time for the Discovery MI and Discovery 690 scanners

All spheres in the NEMA phantom (10–37 mm) were visible at all acquisition times for both scanners. The detectability of the smaller spheres in the Jaszczak phantom can be seen in Fig. 5. The sensitivity was 98% and 88% for the DMI and D690, respectively, for SBR 15:1. For SBR 7.5, the sensitivity was 75% and 83% for the DMI and D690, respectively, and for a SBR of 3.85:1, the sensitivity was 43% and 30% for the DMI and D690, respectively. The logistic mixed models show that there was no statistical difference in the odds for detectability between the two scanners (*p* value 0.075, with a confidence interval of 0.066–1.140).

**Fig. 6:**
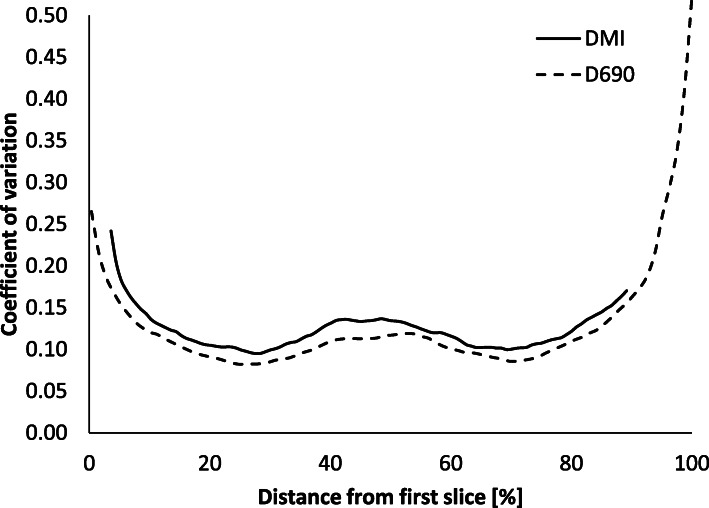
The coefficient of variation (COV) for the Discovery MI and Discovery 690 scanners in two bed positions with an overlap of 24% measured in the homogenous phantom

The homogeneously filled phantom, scanned over two bed positions, showed that the COV is slightly lower for the D690 system. The difference corresponds well with the ratio of the slice thickness, which is equal to 1.17. In the middle of each bed position, the COV reached a minimum level of around 0.10 and, in the overlap, the COV increased to about 0.14 for both systems (Fig. 6). The phantom did not cover the whole axial field of view in the DMI system, rendering the DMI curve not to cover the whole 100% of the FOV.

## Discussion

For large spheres (10–37 mm), no differences between the PET/CT systems could be demonstrated even though the noise level was increased above the level normally seen in patient studies. For the smaller spheres (3.95–9.89 mm), a marginally better detectability at lower noise levels was seen for the SiPM-based scanner.

To best demonstrate differences between the hardware of the systems, the acquisition and reconstruction parameters for the PET-images were the same for both systems. The number of subsets was 16 because this was one of the few choices available that could be the same on both systems. The number of iterations, four, was selected to reach convergence in the reconstruction. No post-filtering was used, but resolution recovery and time-of-flight were used. Although these are scanner specific to some extent, they correct for the specific hardware limitations and are, therefore, part of the system. Another difference that we were unable to influence was the slice thickness.

In all measurements, the noise level was higher for the DMI system. This was because of the smaller voxel size for the DMI (due to the thinner slice thickness, 2.79 and 3.27 mm for the DMI and the D690, respectively). The higher sensitivity of the DMI system depends mainly on the increase in the number of PET-rings from three to four, and not the shift from analogue to digital PM-tubes. Although the inter-block Compton scatter recovery implemented on the DMI may increase the sensitivity by up to 20 %, the study of Vandendriessche et al. on a three-ring DMI system with SiPM showed a sensitivity in the NEMA tests of 7.3 cps/MBq, which is within the same range as the NEMA tests (6.9 cps/MBq) performed on the three-ring system with analogue PM-tubes used in the present study [12]. Although the four-ring DMI sensitivity is approximately double that of the three-ring D690 system, the resulting image noise level is somewhat higher, as demonstrated in Fig. 6. The DMI has a slice thickness of 2.79 mm compared to the D690 thickness of 3.27 mm causing a higher noise level in the DMI images. The DMI images will thus include more noise when using the same OSEM reconstruction parameters.

A patient administrated with 4 MBq/kg, scanned for 1.5 min per position, and where the images are reconstructed with OSEM to convergence and a Gaussian post filter with a full with at half maximum in the range of the spatial resolution, has a noise level, COV, of approximately 0.1 in the liver. Using the same noise level in the phantom (600 s/bed) as for patients gave no differences in detectability between the PET/CT systems. Reconstructions representing shorter acquisition times were, therefore, studied to investigate potential differences at other noise levels.

The slice thickness for the systems is in the same range as the diameter of the smallest spheres that were used in the study. There is, therefore, a risk that the signal and detectability of theses spheres depend on the positioning of the phantom (i.e. if the sphere is positioned in the middle of a detector crystal or between two crystals). However, the measurement was repeated, with the phantom moved 2 mm, with virtually identical results.

The RC-curve for the NEMA Body phantom did not present any apparent difference between the two examined systems. The smaller spheres in the Jaszczak phantom was not evaluated with a recovery curve since the size of the sphere is small in comparison to the spill-out effect and the value would not be correctly reproduced.

The present study demonstrates only a marginal difference between the analogue and SiPM-system that is likely not clinically relevant using OSEM reconstructions. However, there is a theoretical increase in time-of-flight capability with the DMI-system, which is too small to impact the hotspots in the NEMA-body phantom or the smaller hotspots in the Jaszczak phantom, because these phantoms may be too small. Previous studies have reported an increased image quality using BSREM reconstruction algorithms compared to OSEM reconstruction in analogue systems for oncology studies. Higher signal-to-noise ratio/background variability, higher SUV and SUV_max_, and higher signal-to-background ratio/contrast recovery have been reported [9, 13–15]. The same result has been seen on digital SiPM-systems comparing OSEM with BSREM reconstruction [5, 16]. This study indicates that the reported increased image quality with the new digital PET/CT-systems is mainly due to the improved reconstruction algorithm BSREM since no significant differences can be measured for the hardware when comparing the SiPM-system with the modern analogue system from GE Healthcare. Comparing digital and analogue systems from other vendors might give other outcomes if, for example, they have made more changes in crystal size.

SiPM can, however, have other advantages compared to analogue PM-tubes than only the image quality (e.g. higher reliability and longer lifetime). SiPM also provides a higher timing resolution that could be of interest, for example, in absolute quantification of myocardial blood flow.

## Conclusion

Marginally higher contrast in small spheres was seen for the SiPM-based scanner, but no significant difference was found in small lesion detectability between the scanners. The overall performance of the scanners was similar, suggesting that the SiPM-based detectors are not the primary reason for the improved image quality when comparing the analogue and digital PET systems in this study.

## Data Availability

The datasets used and analysed in this study are available from the corresponding author on reasonable request.

## References

[1] Hsu DFC, Ilan E, Peterson WT, Uribe J, Lubberink M, Levin CS (2017). Studies of a next-generation silicon-photomultiplier-based time-of-flight PET/CT system. J Nucl Med.

[2] Roncali E, Cherry SR (2011). Application of silicon photomultipliers to positron emission tomography. Ann Biomed Eng.

[3] Wagatsuma K, Miwa K, Sakata M, Oda K, Ono H, Kameyama M (2017). Comparison between new-generation SiPM-based and conventional PMT-based TOF-PET/CT. Phys Med.

[4] Ross SQ (2014). Clear, GE Healthcare. White paper.

[5] Lindstrom E, Sundin A, Trampal C, Lindsjo L, Ilan E, Danfors T (2018). Evaluation of penalized-likelihood estimation reconstruction on a digital time-of-flight PET/CT scanner for (18)F-FDG whole-body examinations. J Nucl Med.

[6] Nguyen NC, Vercher-Conejero JL, Sattar A, Miller MA, Maniawski PJ, Jordan DW (2015). Image quality and diagnostic performance of a digital PET prototype in patients with oncologic diseases: initial experience and comparison with analog PET. J Nucl Med.

[7] van der Vos CS, Koopman D, Rijnsdorp S, Arends AJ, Boellaard R, van Dalen JA (2017). Quantification, improvement, and harmonization of small lesion detection with state-of-the-art PET. Eur J Nucl Med Mol Imaging.

[8] Zhang J, Maniawski P, Knopp MV (2018). Performance evaluation of the next generation solid-state digital photon counting PET/CT system. EJNMMI Res.

[9] Oddstig J, Leide Svegborn S, Almquist H, Bitzen U, Garpered S, Hedeer F (2019). Comparison of conventional and Si-photomultiplier-based PET systems for image quality and diagnostic performance. BMC Med Imaging.

[10] Salvadori J, Imbert L, Perrin M, Karcher G, Lamiral Z, Marie PY (2019). Head-to-head comparison of image quality between brain (18)F-FDG images recorded with a fully digital versus a last-generation analog PET camera. EJNMMI Res.

[11] Association NEm (2018). NEMA NU 2-2018 performance measurements of positron emission tomographs.

[12] Vandendriessche D, Uribe J, Bertin H, De Geeter F (2019). Performance characteristics of silicon photomultiplier based 15-cm AFOV TOF PET/CT. EJNMMI Phys.

[13] Matti A, Lima GM, Pettinato C, Pietrobon F, Martinelli F, Fanti S (2019). How do the more recent reconstruction algorithms affect the interpretation criteria of PET/CT images?. Nucl Med Mol Imaging.

[14] Teoh EJ, McGowan DR, Bradley KM, Belcher E, Black E, Gleeson FV (2016). Novel penalised likelihood reconstruction of PET in the assessment of histologically verified small pulmonary nodules. Eur Radiol.

[15] Teoh EJ, McGowan DR, Macpherson RE, Bradley KM, Gleeson FV (2015). Phantom and clinical evaluation of the bayesian penalized likelihood reconstruction algorithm Q.Clear on an LYSO PET/CT system. J Nucl Med.

[16] Messerli M, Stolzmann P, Egger-Sigg M, Trinckauf J, D'Aguanno S, Burger IA (2018). Impact of a Bayesian penalized likelihood reconstruction algorithm on image quality in novel digital PET/CT: clinical implications for the assessment of lung tumors. EJNMMI Phys.

